# AI-assisted diplomatic decision-making during crises—Challenges and opportunities

**DOI:** 10.3389/fdata.2023.1183313

**Published:** 2023-05-12

**Authors:** Neeti Pokhriyal, Till Koebe

**Affiliations:** ^1^Department of Computer Science, Dartmouth College, Hanover, NH, United States; ^2^Saarland Informatics Campus, Universität des Saarlandes, Saarbrücken, Germany

**Keywords:** big data, data analytics, artificial intelligence, diplomacy, crisis management, data sharing

## 1. Introduction

When a crisis hits, information is scarce. Who is affected? How is the situation developing? In such adverse events on an international scale, whether the Ebola outbreak in West Africa starting in 2014, the Syrian refugee crisis hitting highs in 2015, the COVID pandemic, or lately the 2022 Russian invasion of Ukraine, countries often depend on diplomatic instruments (such as multilateral engagement, strategic communication, negotiations, and sanctions) and *ad-hoc* intelligence provided by other countries for coordinating actions.

We focus on diplomatic decision-making during crisis times, which we define as theorizing the challenges faced by diplomats and governments during such times and understanding how AI can assist them in monitoring, analyzing, and responding to crises (e.g., armed conflict, migration, pandemic). In that context, we use “AI” as an encompassing term for advanced machine learning methods deployed for classificatory, generative, and predictive tasks. While researchers have, recently, begun to explore digital data and AI-driven analytic tools for assisting in decision-making during crises, a critical concern remains—“Can AI techniques add efficiency to processing information and/or provide insights of significant strategic value that are not possible with traditional means?”

Some of the examples of research in this area are in preparing and conducting diplomatic negotiations, where AI tools have been used for peacebuilding and security in the United Nations (Wählisch, 2020; Masood Alavi et al., 2022). Examples also include intervention planning through computer-assisted strategic reasoning (Bakhtin et al., 2022). AI-assisted tools have been used in providing consular assistance and in better allocating the resources during pandemic times, in managing public expectations (Bjola and Manor, 2020; Bjola, 2022) and in facilitating communication (Arendt-Cassetta, 2021). Other examples include tools based on generative pre-trained models that have been demonstrated to assist diplomats in strategy formation (Stanzel and Voelsen, 2022).

Recent academic works have demonstrated the efficacy of employing or integrating “non-traditional" data (e.g., social media, satellite imagery, etc) for situational awareness tasks. For example, using mobile phone data for pandemic planning (Peak et al., 2018; Nuria, 2020) and for understanding refugee migration (Bruckschen et al., 2019); advertising data from social networks for understanding population dynamics (Leasure et al., 2023), satellite imagery for rapid assessment of displaced populations (Wang, 2015), and aerial imagery for identifying victims (Bravo, 2019), just to name a few.

Despite these successes, we identify four critical challenges unique to the area of diplomacy that needs to be considered within the growing AI and diplomacy community going ahead:

1. First, decisions during crises are almost always taken using limited or incomplete information. There may be deliberate misuse and obfuscation of data/signals between different parties involved. At the start of a crisis, information is usually limited and potentially biased, especially along socioeconomic and rural-urban lines as crises are known to exacerbate the vulnerabilities already existing in the populations. This requires AI tools to quantify and visualize calibrated uncertainty in their outputs in an appropriate manner.
2. Second, in many cases, human lives and livelihoods are at stake. Therefore, any forecast, reasoning, or recommendation provided by AI assistance needs to be explainable and transparent for authorized users, but also secure against unauthorized access as diplomatic information is often highly sensitive. The question of accountability in case of misleading AI assistance needs to be addressed beforehand.
3. Third, in complex situations with high stakes but limited information, cultural differences and value-laden judgment driven by personal experiences play a central role in diplomatic decision-making. This calls for the use of learning techniques that can incorporate domain knowledge and experience.
4. Fourth, diplomatic interests during crises are often multifaceted, resulting in deep mistrust in and strategic misuse of information. Social media data, when used for consular tasks, has been shown to be susceptible to various d-/misinformation campaigns, some by the public, others by state actors for strategic manipulation (Bjola and Manor, 2020).

### 1.1. Contributions

To mitigate the above-mentioned challenges, there is an immediate need to develop a consolidated mechanism for responding to crises using AI-assisted tools and data. In this opinion piece, we put forth three necessary preconditions, discussed below, that should govern the development of newer AI models and the use of digital data for this task:

1. Developing mechanisms for responsible models of data sharing that address the privacy risks across different datasets in a transparent and accountable manner.
2. Designing interpretable AI models that are robust to noise, and generalize well to limited data environments. Ensuring that outputs of AI models take into account biases and communicate uncertainty related to these outputs comprehensively and coherently. We consider this to be a prerequisite for any equitable and fair AI, also beyond the realms of diplomacy.
3. Building capacity of personnel (diplomats, aid organizations, government offices, etc.) who are central during crises to understand the promises and limitations of AI models and their outputs.

Each of the above preconditions is described in detail below. We highlight some examples of research and policy initiatives in other domains for each of these points which could prove relevant to spur innovation in algorithm design for decision-making in diplomacy tasks.

## 2. Responsible models for data sharing that respect privacy

Prioritizing interventions by identifying those most in need is of utmost importance in crisis management. While this is possible with highly granular private data, it raises critical privacy concerns since intervention targeting is often related to (groups of) individuals (De Montjoye et al., 2013; Rocher, 2019). As Mahmood et al. (2010) points out, issues related to data access and processing including identifying potential privacy vulnerabilities need to be addressed ahead of time as an integral part of disaster preparedness in order to avoid both delays in data provisioning and privacy vulnerabilities. However, this becomes more challenging for complex datasets such as those consisting of interaction or mobility or health information.

Recent theoretical advances such as differential privacy (Dwork, 2008), generative modeling (Goodfellow et al., 2020) and secure computation schemes (Sun et al., 2018), but also technological innovations in the area of remote computing such as federated learning (Kairouz et al., 2021) and query-based Q&A systems (Oehmichen et al., 2019) have facilitated privacy-conscious data sharing and have narrowed the void between privacy and utility. However, while residual risks remain (Houssiau et al., 2022), these privacy-enhancing technologies just form the tip of an iceberg.

For responsible data sharing to become reality, it requires talking in a common language. In data, this means spending tremendous efforts on governing, harmonizing, and standardizing definitions and processes, on organizing ongoing consultation rounds and working groups with stakeholders as standards and guidelines are usually *living* documents that need to adapt constantly to a changing landscape. Various consultation processes for standardizing and harmonizing data flows already exist both in the private and the public sector (e.g., the 3GPP on mobile communication protocols or SDMX on statistical (meta-)data exchanges), however, they largely remain silo-ed within their industry. The next step toward true data interoperability would require connecting horizontally between data providers (industries) to move toward creating common data spaces.

While the European Union has set standards with the General Data Protection Regulation (GDPR), the new European Interoperability Framework, and its ambitious GAIA-X project that radiate beyond its own jurisdiction, no common global baseline on responsible data sharing practices yet exists. By looking at data diplomacy on the international level, it appears that this may not realize soon as the controversial discussions on the US-EU Data Privacy Shield (Fioretti and Volz, 2016) and more recently on the Trans-Atlantic Data Privacy Framework (Sawyer, 2022) exemplify.

But as Boyd et al. (2019) points out, data diplomacy is not reserved for state actors but is applicable to the individual as crowdsourcing applications for crises response such as Ushahidi or Facebook's Safety Check showcase.

Designing mechanisms where private data and data products can be shared is an active area of research in the database management community (Stoyanovich et al., 2020). A critical concern, here, is how to incentivize data sharing. As a response, researchers have conceptualized the ideas of data markets (that tackle problems of data sharing, discovery, and integration across heterogeneous data) (Balazinska et al., 2011), data catalogs (that manage metadata) (Subramaniam et al., 2021), data stations (that prioritize human involvement in the data sharing ecosystem) (Xia et al., 2022), data tags (that help navigate legal, privacy and security concerns related to data sharing) (Sweeney et al., 2015), just to name a few.

## 3. Ensuring transparent AI models that respect democratic principles and are robust to noise/missing data

Recent works in automated decision-making have highlighted the issues of bias and discrimination in settings where AI models are trained on (big) datasets that are not representative of the population on which they are asked to make predictions. Models trained on digital data often encode human biases. Prominent studies have shown the bias and discrimination in the algorithmic decision-making for the criminal justice system, money lending, hiring decisions, school admissions, and health care (Mehrabi et al., 2021). However, there is scant research in understanding notions of bias and discrimination when non-traditional data and AI models are used for diplomatic decision-making during crisis times.

Some works reason about demographic bias in the output of AI models employed for humanitarian mapping using satellite imagery (Kondmann and Zhu, 2021) and for targeting aid using mobile phone data (Aiken et al., 2022). Another work by Schlosser et al. (2021) uncovers a significant bias in the mobility data toward wealthy subscribers and demonstrates how it significantly impacts downstream tasks, like mapping the spread of disease.

Beyond reasoning about bias and discrimination, we argue that it is critical that the use of AI and machine learning models trained on non-traditional data do not disproportionately impact vulnerable and marginalized communities, as the structural inequities present within societies may expose these groups to greater risks during any crises. Recent works related to the COVID pandemic have provided evidence of AI's susceptibility to entrench existing health inequities in society (Bolin and Kurtz, 2018; Leslie et al., 2021) and thus amplifying its harm to vulnerable populations. Crisis times put pressure on the policymakers (Mazepus and van Leeuwen, 2020) since a rapid response is demanded. Taking lessons from the pandemic, where AI models were hurriedly repurposed in healthcare (Wynants et al., 2020) and criminal justice settings (Partnership on Artificial Intelligence, 2020), we suggest paying critical attention to guarantees of equity provided by these AI models across different crises scenarios for the planning of appropriate interventions.

Regarding the transparency of AI models, we point out research conceptualizing the use of metadata to support interpretability. Some examples of research in this direction include the use of dataset nutritional labels (analogous to food labels in the food industry) that provide a comprehensive overview of dataset *ingredients* before model development (Holland et al., 2020); datasheets for datasets (analogous to electrical devices) that provide the recommended use of a given dataset (Gebru et al., 2021); model cards that describe how (AI/ML) models are intended to be used (Mitchell et al., 2019); and ranking facts that provide interpretability for the ranked outputs of AI models (Yang et al., 2018). We envision that the use of similar methods for describing metadata associated with datasets that have the potential to be used for diplomatic decision-making would promote transparency and build trust for the broader adoption of such techniques. Analogous to the recent works in clinical benchmarking (Mincu and Roy, 2022), which focus on questions related to the meaningfulness of results derived from using AI/ML models in clinical settings, we suggest conducting studies to understand how well these models assist in diplomatic decision-making when deployed in real-world crises, and what their broader societal implications are.

## 4. Capacity building efforts for an inclusive and sustainable response to crises

The Royal Society and American Association for the Advancement of Science (2010) and similarly Boyd et al. (2019) divide the interplay of diplomacy with science and data, respectively, into three subgroups: data in diplomacy, diplomacy for data, and data for diplomacy. This taxonomy can also be applied to AI and diplomacy.

First, AI supports diplomacy, both through innovative services such as AI-assisted simultaneous translation (Ma, 2019) at international meetings, but also through AI-powered evidence gathering in areas such as climate change (Nishant et al., 2020), conservation (Wearn et al., 2019), and drug discovery (Jiménez-Luna et al., 2020). However, communicating these findings appropriately requires a common understanding of the respective processes. Collaborative efforts such as the Intergovernmental Panel on Climate Change (IPCC) exemplify the complexity and the organizational challenges of translating scientific evidence into policy guidance on a global scale.

Second, diplomacy is required to reconcile technological innovations related to AI with laws and ethics by negotiating appropriate (international) governance frameworks. This, however, requires a basic understanding of the important concepts, priorities, and needs in that area by relevant personnel. Multi-national initiatives such as the Global Partnership on Artificial Intelligence (GPAI) (OECD, 2020) foster such skills and connect government officials to stakeholders from academia, civil society, and the private sector and help to steer research funding to priority areas. The so-called “Facebook files"—revelations about the role of vastly unregulated social networks in inciting violence around the world by amplifying hate speech, among other things (Horwitz, 2021)—provide an arguable example of failing to address governance needs in AI-assisted technologies. Thus, further educating diplomats and other relevant government officials about the opportunities and potential impacts of AI-powered technologies can be regarded as a risk-reduction measure. Furthermore, AI, being regarded as a key technology of the future, requires diplomatic efforts on its own as countries try to defend or gain a technological advantage in that area through protectionist measures (Coca, 2019). For AI not to become an accelerator of global inequalities, bi- or multilateral diplomacy is needed to address these concerns of technology protectionism while protecting open research opportunities.

Third, as Sakurai and Murayama (2019) stresses, while public disaster management plans usually include business continuity plans for relevant infrastructure including ICTs, little guidance has long been available to public officials on how to use these technologies for situational awareness and decision-making for crisis response. International organizations such as UN Global Pulse and UN OCHA, private companies such as Palantir, and collaborative science efforts such as the Data for Refugee challenge (Salah et al., 2018) or the joint U.S–U.K. Prize Challenges (UK Centre for Data Ethics & Innovation et al., 2022) showcase recent advances toward building collaborations and partnerships among multiple stakeholders at a global scale that include funding agencies, industries, and governments in designing technologies for a coherent response during crises. At the same time, AI systems are shown to increasingly become better at human-like conversations and strategic reasoning (Bakhtin et al., 2022), thus potentially altering the way how negotiations, training, and simulation exercises in preparation for or during the crisis are conducted in the future.

## 5. Conclusion

Our position paper identifies three challenges specific to the field of AI and diplomacy, namely decision-making using limited information characterized by scenarios where mistrust and strategic misuse of information is often prevalent; where the stakes are high and resources are constrained; and where there is an imminent need to integrate data-driven insights with the value-laden judgment of diplomats. To address these challenges and unearth synergies in this field, especially during crisis times, we put forth three critical preconditions. These are data sharing while respecting privacy; ensuring transparent AI models that are robust to noise/missing data, and building capacity and collaborations for sustained global cooperation not only to propel technical innovation and align regulations but, more importantly, to engage diplomats, governments, and communities who should reap the most benefits of these technologies.

However, there are no simple solutions for this as these preconditions are still active areas of research and will eventually not be fulfilled in the foreseeable future. For example, with the complexity of large language models challenging explainability in the short-term, regulating the quality of training data in combination with liability requirements of AI-based service providers may act as a workaround to ensure that the technology is safe and the output trustworthy. Furthermore, quantifying uncertainty accurately and achieving robustness holistically may be out of reach in the field of diplomacy any time soon, however, creating a code of practice similar to the ISO/IEC 27000-series for information security may help to make headway on AI-assisted diplomatic decision-making. Finally, data sharing remains in too many countries a regulatory gray area as relevant frameworks on data privacy are non-existent or outdated. As a consequence, markets for non-traditional data tend to be opaque with nontransparent price-setting mechanisms and black box preprocessing. This negatively affects the legitimacy and trustworthiness of any knowledge generated thereof. Harmonizing and updating regulatory frameworks and defining rules for technological products and services depending on the level of expected societal impact could allow for better products and services while at the same time improving their economies of scale. These rules include but are not limited to regular auditing, extensive testing, and detailed documentation of algorithms and datasets. The European AI Act provides a precedent in this direction. Even though these efforts should be global, the past has shown that regional efforts might evolve to de-facto standards informally as seen with the European General Data Protection Regulation or more recently with the European AI Act. Finally, drawing on a lesson from disaster risk management, i.e., that only systems that are used on a frequent basis will also be used in times of crisis, this effectively calls for the integration of AI assistance into diplomatic business processes at large.

## Author contributions

NP and TK contributed substantially to the conception of the work, drafted and revised the content, provided approval for the publication of the content, and agree to be accountable for all aspects of the work in ensuring that questions related to the accuracy or integrity of any part of the work are appropriately investigated and resolved. All authors contributed to the article and approved the submitted version.

## References

[B1] Aiken E. Bellue S. Karlan D. Udry C. Blumenstock J. E. (2022). Machine learning and phone data can improve targeting of humanitarian aid. Nature 603, 864–870. 10.1038/s41586-022-04484-9 35296856 PMC8967719

[B2] Arendt-Cassetta L. (2021). From Digital Promise to Frontline Practice: New and Emerging Technologies in Humanitarian Action. New York, NY: OCHA.

[B3] Bakhtin A. Brown N. Dinan E. Farina G. Flaherty C. Fried D.. (2022). Human-level play in the game of *Diplomacy* by combining language models with strategic reasoning. Science 378, 1067–1074. 10.1126/science.ade9097 36413172

[B4] Balazinska M. Howe B. Suciu D. (2011). Data markets in the cloud: an opportunity for the database community. Proc. VLDB Endow. 4, 1482–1485. 10.14778/3402755.3402801

[B5] Bjola C. (2022). Artificial Intelligence and Diplomatic Crisis Management: Addressing the ‘Fog of War’. Oxford: Working Paper No 6. Oxford Digital Diplomacy Research.

[B6] Bjola C. Manor I. (2020). Digital Diplomacy in the Time of the Coronavirus Pandemic. Los Angeles, CA: USC Center on Public Diplomacy Blog. p. 31.

[B7] Bolin B. Kurtz L. C. (2018). Race, class, ethnicity, and disaster vulnerability, in Handbook of Disaster Research, eds Rodrguez H. Trainor J. E. Donner W. R. (New York, NY: Springer), 181–203. 10.1007/978-3-319-63254-4_10

[B8] Boyd A. Gatewood J. Thorson S. Dye T. D. (2019). Data diplomacy. Sci. Dipl. 8. PMC6785044 31598426

[B9] Bravo R. Z. B. (2019). The use of uavs in humanitarian relief: an application of pomdp-based methodology for finding victims. Prod. Oper. Manag. 28, 421–440. 10.1111/poms.12930

[B10] Bruckschen F. Koebe T. Ludolph M. Marino M. F. Schmid T. (2019). Refugees in undeclared employment “a case study in turkey, in Guide to Mobile Data Analytics in Refugee Scenarios, eds Ali Salah A. Pentland A. Lepri B. Letouz E. (Cham: Springer), 329–346. 10.1007/978-3-030-12554-7_17

[B11] Coca N. (2019). Chinas Digital Protectionism Puts the Future of the Global Internet at Risk. Washington, DC: The Washington Post.

[B12] De Montjoye Y.-A. Hidalgo C. A. Verleysen M. Blondel V. D. (2013). Unique in the crowd: the privacy bounds of human mobility. Sci. Rep. 3, 1–5. 10.1038/srep01376 23524645 PMC3607247

[B13] Dwork C. (2008). Differential privacy: a survey of results, in, *Theory and Applications of Models of Computationed*, ed Agrawal M. (Cham: Springer), 1–19. 10.1007/978-3-540-79228-4_1

[B14] Fioretti J. Volz D. (2016). Privacy Group Launches Legal Challenge Against EU-U.S. Data Pact. London: Reuters.

[B15] Gebru T. Morgenstern J. Vecchione B. Vaughan J. W. Wallach H. Iii H. D.. (2021). Datasheets for datasets. Commun. ACM, 64, 86–92. 10.1145/3458723

[B16] Goodfellow I. Pouget-Abadie J. Mirza M. Xu B. Warde-Farley D. Ozair S.. (2020). Generative adversarial networks. Commun. ACM, 63, 139–144. 10.1145/3422622

[B17] Holland S. Hosny A. Newman S. Joseph J. Chmielinski K. (2020). The dataset nutrition label. Data Prot. Priv. 12, 1. 10.5040/9781509932771.ch-001

[B18] Horwitz J. (2021). The Facebook Files. New York, NY: The Wall Street Journal.

[B19] Houssiau F. Rocher L. de Montjoye Y.-A. (2022). On the difficulty of achieving differential privacy in practice: user-level guarantees in aggregate location data. Nat. Commun. 13, 1–3. 10.1038/s41467-021-27566-0 35013315 PMC8748776

[B20] Jiménez-Luna J. Grisoni F. Schneider G. (2020). Drug discovery with explainable artificial intelligence. Nat. Mach. Intell. 2, 573–584. 10.1038/s42256-020-00236-4

[B21] Kairouz P. McMahan H. B. Avent B. Bellet A. Bennis M. Bhagoji A. N.. (2021). Advances and open problems in federated learning. Found. Trends Mach. Learn. 14, 1–210. 10.1561/9781680837896

[B22] Kondmann L. Zhu X. X. (2021). Under the radar-auditing fairness in ml for humanitarian mapping. arXiv. [preprint]. 10.48550/arXiv.2108.02137

[B23] Leasure D. R. Kashyap R. Rampazzo F. Dooley C. A. Elbers B. Bondarenko M.. (2023). Nowcasting Daily Population Displacement in Ukraine Through Social Media Advertising Data. Population and Development Review. 10.1111/padr.12558

[B24] Leslie D. Mazumder A. Peppin A. Wolters M. K. Hagerty A. (2021). Does AI stand for augmenting inequality in the era of covid-19 healthcare? BMJ 372, 3837493. 10.2139/ssrn.3837493 33722847 PMC7958301

[B25] Ma M. (2019). Stacl: simultaneous translation with implicit anticipation and controllable latency using prefix-to-prefix framework, in Proceedings of the 57th Annual Meeting of the Association for Computational Linguistics (Florence, Italy), 3025–3036. 10.18653/v1/P19-1289

[B26] Mahmood J. Ngom M. Delargy P. Tambashe B. Jongstra E. Oussein S.. (2010). Guidelines on Data Issues in Humanitarian Crisis Situations. Technical report. New York, NY: UNFPA.

[B27] Masood Alavi D. Wählisch M. Irwin C. Konya A. (2022). Using artificial intelligence for peacebuilding. J. Peacebuilding Dev. 17, 239–243. 10.1177/15423166221102757

[B28] Mazepus H. van Leeuwen F. (2020). Fairness matters when responding to disasters: An experimental study of government legitimacy. Governance 33, 621–637. 10.1111/gove.12440

[B29] Mehrabi N. Morstatter F. Saxena N. Lerman K. Galstyan A. (2021). A survey on bias and fairness in machine learning. ACM Comput. Surv. 54, 1–35. 10.1145/3457607

[B30] Mincu D. Roy S. (2022). Developing robust benchmarks for driving forward ai innovation in healthcare. Nat. Mach. Intell. 4, 1–6. 10.1038/s42256-022-00559-4

[B31] Mitchell M. Wu S. Zaldivar A. Barnes P. Vasserman L. Hutchinson B.. (2019). Model cards for model reporting, in Proceedings of the Conference on Fairness, Accountability, and Transparency (New York, NY), 220–229. 10.1145/3287560.3287596

[B32] Nishant R. Kennedy M. Corbett J. (2020). Artificial intelligence for sustainability: Challenges, opportunities, and a research agenda. Int. J. Inf. Manag. 53, 102104. 10.1016/j.ijinfomgt.2020.102104

[B33] Nuria O. (2020). Mobile phone data for informing public health actions across the covid-19 pandemic life cycle. Sci. Adv. 6, eabc0764. 10.1126/sciadv.abc0764 32548274 PMC7274807

[B34] OECD. (2020). Global Partnership on Artificial Intelligence. Available online at: https://gpai.ai/ (accessed April 27, 2023).

[B35] Oehmichen A. Jain S. Gadotti A. de Montjoye Y.-A. (2019). Opal: high performance platform for large-scale privacy-preserving location data analytics, in 2019 IEEE International Conference on Big Data (Big Data) (Los Angeles, CA: IEEE), 1332–1342. 10.1109/BigData47090.2019.9006389

[B36] Partnership on Artificial Intelligence (2020). Global Partnership on Artificial Intelligence.

[B37] Peak C. M. Wesolowski A. zu Erbach-Schoenberg E. Tatem A. J. Wetter E. Lu X.. (2018). Population mobility reductions associated with travel restrictions during the ebola epidemic in sierra leone: use of mobile phone data. Int. J. Epidemiol. 47, 1562–1570. 10.1093/ije/dyy095 29947788 PMC6208277

[B38] Rocher L. (2019). Estimating the success of re-identifications in incomplete datasets using generative models. Nat. Commun. 10, 1–9. 10.1038/s41467-019-10933-3 31337762 PMC6650473

[B39] Sakurai M. Murayama Y. (2019). Information technologies and disaster management-benefits and issues. Prog. Disaster Sci. 2, 100012. 10.1016/j.pdisas.2019.100012

[B40] Salah A. A. Pentland A. Lepri B. Letouzé E. Vinck P. de Montjoye Y.-A.. (2018). Data for refugees: the d4r challenge on mobility of Syrian refugees in Turkey. arXiv. [preprint]. 10.48550/arXiv.1807.00523

[B41] Sawyer K. (2022). Does the latest move in trans-Atlantic privacy really change the game? The National Law Review, November 16, 2022.

[B42] Schlosser F. Sekara V. Brockmann D. Garcia-Herranz M. (2021). Biases in human mobility data impact epidemic modeling. arXiv. [preprint]. 10.48550/arXiv.2112.12521

[B43] Stanzel V. Voelsen D. (2022). Diplomacy and Artificial Intelligence: Reflections on Practical Assistance for Diplomatic Negotiations, Vol. 1/2022 of SWP Research Paper. Berlin: Stiftung Wissenschaft und Politik -SWP- Deutsches Institut für Internationale Politik und Sicherheit. 10.18449/2022RP01

[B44] Stoyanovich J. Howe B. Jagadish H. (2020). Responsible data management. Proc. VLDB Endowment 13, 3474–3488. 10.14778/3415478.3415570

[B45] Subramaniam P. Ma Y. Li C. Mohanty I. Fernandez R. C. (2021). Comprehensive and comprehensible data catalogs: the what, who, where, when, why, and how of metadata management. arXiv. [preprint]. 10.48550/arXiv.2103.07532

[B46] Sun X. Zhang P. Liu J. K. Yu J. Xie W. (2018). Private machine learning classification based on fully homomorphic encryption. IEEE Trans. Emerg. Topics Comput. 8, 352–364. 10.1109/TETC.2018.2794611

[B47] Sweeney L. Crosas M. Bar-Sinai M. (2015). Sharing sensitive data with confidence: The datatags system. Technol. Sci. 2015101601. Available online at: http://techscience.org/a/2015101601

[B48] The Royal Society American Association for the Advancement of Science. (2010). New Frontiers in Science Diplomacy. The Royal Society. Available online at: https://www.aaas.org/sites/default/files/New_Frontiers.pdf

[B49] UK Centre for Data Ethics & Innovation, Innovate UK, U.S. National Institute of Standards and Technology, the U.S. National Science Foundation, and White House Office of Science and Technology Policy. (2022). U.K.- U.S. Prize Challenges - Accelerating the Adoption and Development of Privacy-Enhancing Technologies (Pets). Available online at: https://petsprizechallenges.com/ (accessed April 27, 2023).

[B50] Wählisch M. (2020). Big data, new technologies, and sustainable peace: Challenges and opportunities for the un. J. Peacebuilding Dev. 15, 122–126. 10.1177/1542316619868984

[B51] Wang S. (2015). Detecting tents to estimate the displaced populations for post-disaster relief using high resolution satellite imagery. Int. J. Appl. Earth Obs. Geoinf. 36, 87–93. 10.1016/j.jag.2014.11.013

[B52] Wearn O. R. Freeman R. Jacoby D. M. (2019). Responsible ai for conservation. Nat. Mach. Intell. 1, 72–73. 10.1038/s42256-019-0022-7

[B53] Wynants L. Van Calster B. Collins G. S. Riley R. D. Heinze G. Schuit E.. (2020). Prediction models for diagnosis and prognosis of covid-19: systematic review and critical appraisal. BMJ 369, m1328. 10.1136/bmj.m1328 32265220 PMC7222643

[B54] Xia S. Zhu Z. Zhu C. Zhao J. Chard K. Elmore A. J.. (2022). Data station: delegated, trustworthy, and auditable computation to enable data-sharing consortia with a data escrow. Proc. VLDB Endowment 15, 3172–3185. 10.14778/3551793.3551861

[B55] Yang K. Stoyanovich J. Asudeh A. Howe B. Jagadish H. Miklau G.. (2018). A nutritional label for rankings, in Proceedings of the 2018 International Conference on Management of Data (New York, NY), 1773–1776. 10.1145/3183713.3193568

